# Modeling complex genetic and environmental influences on comorbid bipolar disorder with tobacco use disorder

**DOI:** 10.1186/1471-2350-11-14

**Published:** 2010-01-26

**Authors:** Richard C McEachin, Nancy L Saccone, Scott F Saccone, Yelena D Kleyman-Smith, Tiara Kar, Rajesh K Kare, Alex S Ade, Maureen A Sartor, James D Cavalcoli, Melvin G McInnis

**Affiliations:** 1 Department of Psychiatry, University of Michigan, Ann Arbor, MI, USA; 2 National Center for Integrative Biomedical Informatics, University of Michigan, Ann Arbor, MI, USA; 3 Department of Genetics, Washington University, Saint Louis, MO, USA; 4 Department of Psychiatry, Washington University, Saint Louis, MO, USA; 5 Department of Biology, Eastern Michigan University, Ypsilanti, MI, USA

## Abstract

**Background:**

Comorbidity of psychiatric and substance use disorders represents a significant complication in the clinical course of both disorders. Bipolar Disorder (BD) is a psychiatric disorder characterized by severe mood swings, ranging from mania to depression, and up to a 70% rate of comorbid Tobacco Use Disorder (TUD). We found epidemiological evidence consistent with a common underlying etiology for BD and TUD, as well as evidence of both genetic and environmental influences on BD and TUD. Therefore, we hypothesized a common underlying *genetic *etiology, interacting with nicotine exposure, influencing susceptibility to both BD and TUD.

**Methods:**

Using meta-analysis, we compared TUD rates for BD patients and the general population. We identified candidate genes showing statistically significant, replicated, evidence of association with both BD and TUD. We assessed commonality among these candidate genes and hypothesized broader, multi-gene network influences on the comorbidity. Using Fisher Exact tests we tested our hypothesized genetic networks for association with the comorbidity, then compared the inferences drawn with those derived from the commonality assessment. Finally, we prioritized candidate SNPs for validation.

**Results:**

We estimate risk for TUD among BD patients at 2.4 times that of the general population. We found three candidate genes associated with both BD and TUD (COMT, SLC6A3, and SLC6A4) and commonality analysis suggests that these genes interact in predisposing psychiatric and substance use disorders. We identified a 69 gene network that influences neurotransmitter signaling and shows significant over-representation of genes associated with BD and TUD, as well as genes differentially expressed with exposure to tobacco smoke. Twenty four of these genes are known drug targets.

**Conclusions:**

This work highlights novel bioinformatics resources and demonstrates the effectiveness of using an integrated bioinformatics approach to improve our understanding of complex disease etiology. We illustrate the development and testing of hypotheses for a comorbidity predisposed by both genetic and environmental influences. Consistent with our hypothesis, the selected network models multiple interacting genetic influences on comorbid BD with TUD, as well as the environmental influence of nicotine. This network nominates candidate genes for validation and drug testing, and we offer a panel of SNPs prioritized for follow-up.

## Background

Bipolar Disorder (BD) is a severe psychiatric disorder, characterized by periods of mania and depression, which affects approximately 1% of the U.S. population, or 3 - 5% if BD spectrum disorders (BPII and BP-NOS) are included [1,2]. Tobacco Use Disorder (TUD) is the single greatest cause of preventable death in the United States [3,4] and it disproportionately affects psychiatric patients [5]. Note that TUD is defined as "Tobacco used to the detriment of a person's health or social functioning. Tobacco dependence is included.", according to the Medical Subject Heading (MeSH) index [6]. In earlier work, various research groups used "nicotine dependence", "nicotine addiction", "tobacco dependence", or "smoking" to characterize the phenotype, but there is no clear standard term in the literature. We use the MeSH term, TUD, in these analyses because it incorporates the information derived from these multiple sources and facilitates the bioinformatics analyses. There is evidence of increased risk for TUD among BD patients [1,7-12] as well as evidence that smokers may be at increased risk for BD [1,9]. While it is possible that TUD could predispose individuals to BD, or BD could predispose individuals to TUD, the observed bi-directional increased risk for both disorders is consistent with some common underlying etiology for BD and TUD.

There is epidemiological evidence of multiple genetic influences on both BD [13] and TUD [14]. In family and twin studies, heritability of BD is estimated at 75 to 85% [15,16], consistent with one or more genetic influences on BD susceptibility. Still, even for monozygotic twins, concordance is estimated at only about 67% [17], consistent with environmental influences on BD susceptibility. Heritability of TUD is estimated at 37 to 56% for initiation of smoking, and 59 to 70% for transition to nicotine dependence [18,19], consistent with one or more genetic influences on TUD susceptibility. In addition, nicotine binds to cell surface nicotinic acetylcholine receptors, and so represents an environmental effect which would be expected to influence intracellular signal transduction pathways. Given the potential for some common underlying etiology for BD and TUD, as well as evidence of genetic and environmental influences on both BD and TUD, we hypothesized a common underlying *genetic *etiology, interacting with environmental nicotine exposure, influencing susceptibility for both BD and TUD.

**Figure 1:**
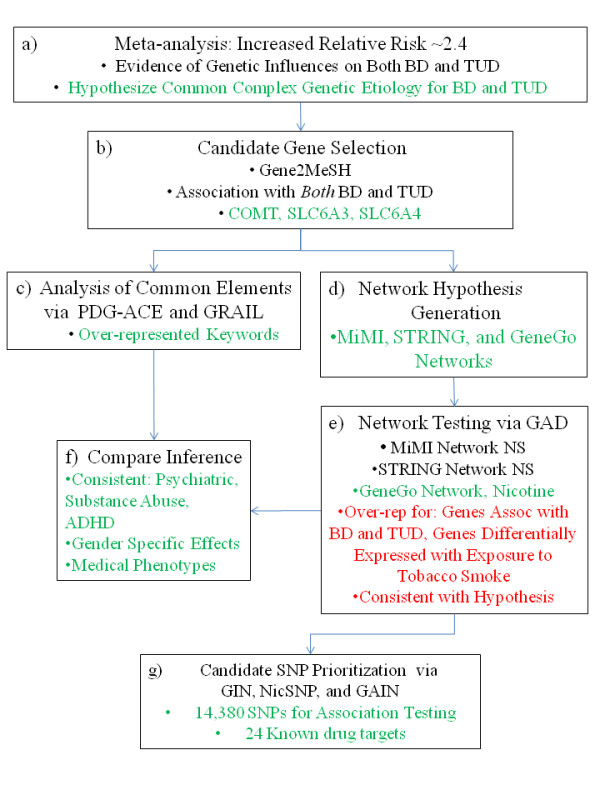
**analysis flow**. Analysis proceeds from meta-analysis, to hypothesis generation, to candidate gene selection, common elements assessment, network hypothesis generation, hypothesis testing, comparison of results, and SNP prioritization.

Figure 1 outlines the overall analysis flow. After assessing the strength of evidence for comorbidity of BD and TUD via meta-analysis, and seeing an increased Relative Risk consistent with some common etiology, we identified candidate genes for the comorbidity. The analysis then followed two parallel paths. First, in complex diseases multiple genetic influences converge on a single phenotype (in this case, co-morbid BD with TUD). We presume that to influence a single phenotype these multiple genetic influences must impact some common element(s) associated with the phenotype (e.g., a common pathway, tissue, cellular function, disease or other process). We exploited this convergence on a single phenotype by identifying significant commonality among the selected candidate genes. We then used this commonality to improve our understanding of the roles of these genes in both BD and TUD. In a parallel analysis, we generated networks of genes that interact with our selected candidate genes. Based on these interactions, we hypothesized models of the larger set of genetic influences on co-morbid BD with TUD, then tested each of these hypotheses for enrichment of BD and TUD associated genes. As with the commonality analysis, analysis of gene networks enriched for BD and TUD associated genes may improve our understanding of the comorbidity, so we compared lessons learned in the commonality and network analyses. Finally, while no GWA studies have yet been conducted specifically to identify candidate genes for this co-morbidity, we prioritized Single Nucleotide Polymorphisms (SNPs) for follow-on studies by functional data, as well as by combining evidence from two GWA studies (one for BD and one for TUD).

## Methods

### Meta-analysis (Figure 1a)

We first assessed the strength of evidence for comorbidity of BD and TUD, based on the published literature. Querying all of PubMed, we found seven studies published on the subject between 1986 and 2008 [1,7-12]. From each study, we selected the data specific to comorbid BD with TUD and ensured that the phenotypes studied were consistent, then generated a combined spreadsheet of the raw data (Additional file 1, Forest Notes). Using MIX Meta-Analysis software [20,21] (version 1.7), we generated an annotated forest plot of Relative Risk, using first a fixed effects model with Mantel-Haenszel weighting then a random effects model with DerSimonian-Laird weighting. All other MIX parameters were set at the default values, under the Analysis/preferences tab. For details of the meta-analysis see Additional file 2, MOOSE Checklist [22].

### Candidate gene selection (Figure 1b)

We used Gene2MeSH [23], a novel bioinformatics resource from the National Center for Integrative Biomedical Informatics (NCIBI), to select candidate genes for comorbid BD with TUD on 8 April, 2009. Gene2MeSH identifies genes and MeSH terms that co-occur in PubMed-indexed manuscripts that are annotated for both the genes referenced and the MeSH terms assigned. Gene2MeSH allows the user to input a MeSH term and find all genes significantly over-represented in publications annotated for that term. In this mode, Gene2MeSH requires MeSH terms as input and we used the MeSH database [6] to identify appropriate MeSH terms to query Gene2MeSH for genes related to BD and TUD. MeSH has only one term for BD, "bipolar disorder", which we used in the Gene2MeSH query to identify a preliminary set of human BD candidate genes. In addition to TUD, MeSH has two related terms so we queried "tobacco use disorder", "nicotine" and "smoking", then used the union of these three human gene sets as a preliminary set of TUD candidate genes.

Gene2Mesh generates a Fisher Exact p-value to quantify the over-representation of genes occurring in publications annotated for a given MeSH term, relative to all papers in PubMed, and select genes based on a threshold of Fisher Exact p-value ≤ 10^-4^. However, co-occurrence of genes with MeSH terms is not the same as association. We tested each preliminary candidate gene for evidence of association between the gene and the appropriate phenotype (i.e., BD or TUD) by reading the papers cited by Gene2MeSH. We accepted in our final "overlapping" set only those preliminary candidate genes for which we found *at least two studies that showed statistically significant positive association (Bonferroni corrected p-value ≤ 0.05) with both BD and TUD*, in the peer reviewed literature. We did not consider power, as this parameter is generally not reported in the literature.

### Common elements (Figure 1c)

In complex diseases, multiple genetic influences converge on a single phenotype, consistent with some common element(s) among these genetic influences (e.g., a common disease process, metabolic or signaling pathway, cellular component, or tissue expression). Understanding commonality among our candidate genes may yield useful inference on how multiple genetic influences converge on the co-morbidity. We assessed two resources available for commonality testing: PDG-ACE (Prioritizing Disease Genes by Analysis of Common Elements) [24,25] from NCIBI, and GRAIL (Gene Relationships Across Implicated Loci) [26] from the Broad Institute. Both PDG-ACE and GRAIL generate hypotheses on gene-gene interactions and also provide quantitative measures of the strength of evidence in support of each hypothesis.

PDG-ACE identifies significant commonality across genetic loci based on text in the Entrez Gene records of genes at locus pairs. We submitted our set of three overlapping candidates to PDG-ACE in pairs, performed 10^7 ^iterations for significance testing using PDG-ACE's MeSH-derived controlled vocabulary of 2,531 keywords, applied a Bonferroni correction for these 2,531 hypothesis tests, and stored the keywords that were significantly over-represented (corrected p-value ≤ 0.05) at each locus pair. For these stored keywords, we assessed the context of each keyword in the Entrez Gene records for each locus and retained those keywords that were used in the same context at both loci. Since the Entrez Gene records for these genes include links to the PubMed abstracts, we followed each of these links to identify trends that may be useful in understanding the roles of these genes in co-morbid BD with TUD.

GRAIL also finds commonality among genes, though, with GRAIL commonality is based on PubMed abstracts. On 8 July, 2009, we input our overlapping candidates to the GRAIL server and set the query regions to equal the seed regions, as recommended in the GRAIL FAQs when the number of input genes is small. We compared the returned keywords associating the overlapping candidate genes to our PDG-ACE results and assessed their context with respect to comorbid BD with TUD.

### Hypothesis generation: network model building (Figure 1d)

We assessed three resources for building models of genetic interactions among our candidate genes: MiMI (Michigan Molecular Interactions) from NCIBI [27], STRING (Search Tool for the Retrieval of Interacting Genes/Proteins) from the European Molecular Biology Lab [28,29], and MetaCore [30] from GeneGo Inc. Each of the models developed using these resources represents one hypothesis on how multiple genetic variants could interact to influence the comorbidity. To focus on the interactions most closely tied to the overlapping candidate genes (and, we assume, most likely to influence comorbid BD with TUD), we set input parameters to accept only the highest quality interactions data and to build the smallest network that includes all of the overlapping candidate genes in a single model.

MiMI includes comprehensive protein interaction information that has been integrated and merged from diverse protein interaction databases. For input of multiple genes, MiMI is implemented as a plug-in for Cytoscape [31] (version 2.6.0), an open source bioinformatics platform for visualizing molecular interaction networks. MiMI does not have a parameter for selecting the level of confidence for interactions. We input our list of three overlapping candidate genes and selected the "Interactions among query genes" option. This network did not include all three of the overlapping candidates in a single network so we moved to the "Query Genes + Nearest Neighbors" option and this produced a single network including all three overlapping candidates. We downloaded the network in both graphical and text formats.

STRING is a database of known and predicted protein interactions including: direct (physical) and indirect (functional) associations derived from genomic context, high-throughput experiments, conserved co-expression, and publications. We input our list of overlapping candidate genes, set the minimum combined score to 0.900 (highest confidence) and built the network. The resulting network did not connect the three overlapping candidate genes, so we had STRING add nodes to the network, one at a time, until all of the overlapping candidate genes were included in a single network. We downloaded the network in both graphical and text formats.

MetaCore (GeneGo Inc.) is a commercial database of human curated data on gene-gene, gene-DNA, and gene-small molecule interactions. The types of interactions data available are equivalent to those available in STRING. Starting with our three overlapping candidates, we set parameters for the "Shortest Paths" network building algorithm and "curated only" data, accepting unspecified effects as well as functional and binding interactions (MetaCore version 6.0). We first looked for direct interactions, then increased the number of nodes allowed between the overlapping candidate genes until they were all included in a single network. The resulting network is comparable to the MiMI and STRING networks.

An important feature of GeneGo, not yet available in MiMI or STRING, is that it allows the user to add selected nodes (genes, small molecules, etc.) to an established network. To help assess the environmental impact of nicotine on the hypothesized network for comorbid BD with TUD, we added nicotine to the network. GeneGo has a built in test for over-representation of genes in documented pathways, so we tested the network for pathway association. We downloaded both GeneGo networks, one excluding nicotine and one including nicotine, in both graphical and text formats.

### Hypothesis testing (Figure 1e)

For each of our hypothesized networks, we first used the Genetic Association Database (GAD) [32] via the DAVID interface (Database for Annotation, Visualization and Integrated Discovery) [33,34], to test for over-representation of genes associated with BD and TUD. In addition, since nicotine represents an environmental influence on our hypothesized networks and differential gene expression is one of the most important ways that cells respond to the environment, we used NCIBI's ConceptGen [35] software application to test for differential gene expression related to BD and/or TUD.

GAD is an archive of results from human genetic association studies of complex diseases and disorders, which has been made available for assessment of gene sets via DAVID. If, based on GAD data, a given network is over-represented for genes associated with both BD and TUD, the network may lead us to a clearer understanding of how the multiple genetic influences converge on the comorbidity. GAD provides dichotomous annotation of genes, based on published evidence, where each gene either has shown evidence or has not (yet) shown evidence of association with a specific phenotype (e.g. BD or TUD). DAVID uses this dichotomous annotation in a modified Fisher Exact test, where the count of positive agreement is reduced by 1 to make a more conservative test, to assess gene sets for over-representation of genes annotated for specific phenotypes. For each of the gene sets nominated by our network building tools (MiMI, STRING, and two GeneGo networks), we set DAVID to assess GENETIC_ASSOCIATION_DB_DISEASE functional annotation. The resulting tables provide test data including the False Discovery Rates (FDR) for specific disease phenotypes. We set FDR ≤ 5% as the threshold for over-representation of genes for any GAD phenotype. Each network, as a whole, may be over-represented for genes associated with BD and/or TUD. However, each of these networks includes the three overlapping candidates, which are already documented to be associated with both BD and TUD. As such, we tested each network a second time, excluding the overlapping candidates. The first test for each network serves as a positive control, where we expect to find evidence in GAD supporting association with BD and TUD. The second test, excluding the overlapping candidates, tests whether the network provides significant new information on association with BD and/or TUD, beyond the influence of the overlapping candidates.

In a second phase of hypothesis testing, we used ConceptGen [35] to test the genes in the larger GeneGo network for over-representation of genes differentially expressed with nicotine exposure. ConceptGen uses a custom-built analysis pipeline for processing Affymetrix GEO [36] datasets from raw data, testing for differentially expressed genes [37], then building concepts to represent the expression profiles. ConceptGen assesses over-representation of gene groups for given concepts by enrichment testing, using the same modified Fisher Exact test as in DAVID. After seeing significant over-representation of genes associated with BD and TUD in the GeneGo network that includes nicotine, we queried ConceptGen for "Gene Expression" concepts that show over-representation of the genes in this network. As in our GAD analysis, we set a threshold of FDR ≤ 0.05 for over-representation of any differentially expressed gene set. (Note that ConceptGen presents FDR as a decimal value while GAD presents FDR as a percentage.)

### Inference from common elements versus network model building (Figure 1f)

The common elements analysis is based on only the overlapping candidate genes, while the network model building analysis includes additional candidate genes. Since these results are related, we compared the inference that could be drawn from the two approaches.

### Prioritizing candidate SNPs for follow-on testing via GIN (Figure 1g)

We first prioritized SNPs in and near the genes in our selected network via the Genomic Information Network (GIN) method developed by Saccone et al. [38]. GIN prioritizes SNPs based on biological relevance, as determined by SNP/gene functional properties including synonymy, annotation for promoter regions, and human/mouse evolutionary conservation. Second, while there are not yet any published GWAS results for co-morbid BD with TUD, we further prioritized SNPs by weighting them based on evidence from the NicSNP GWA [39,40] study of nicotine dependence and the GAIN GWA study of BD[41].

## Results

### Meta-analysis

**Figure 2:**
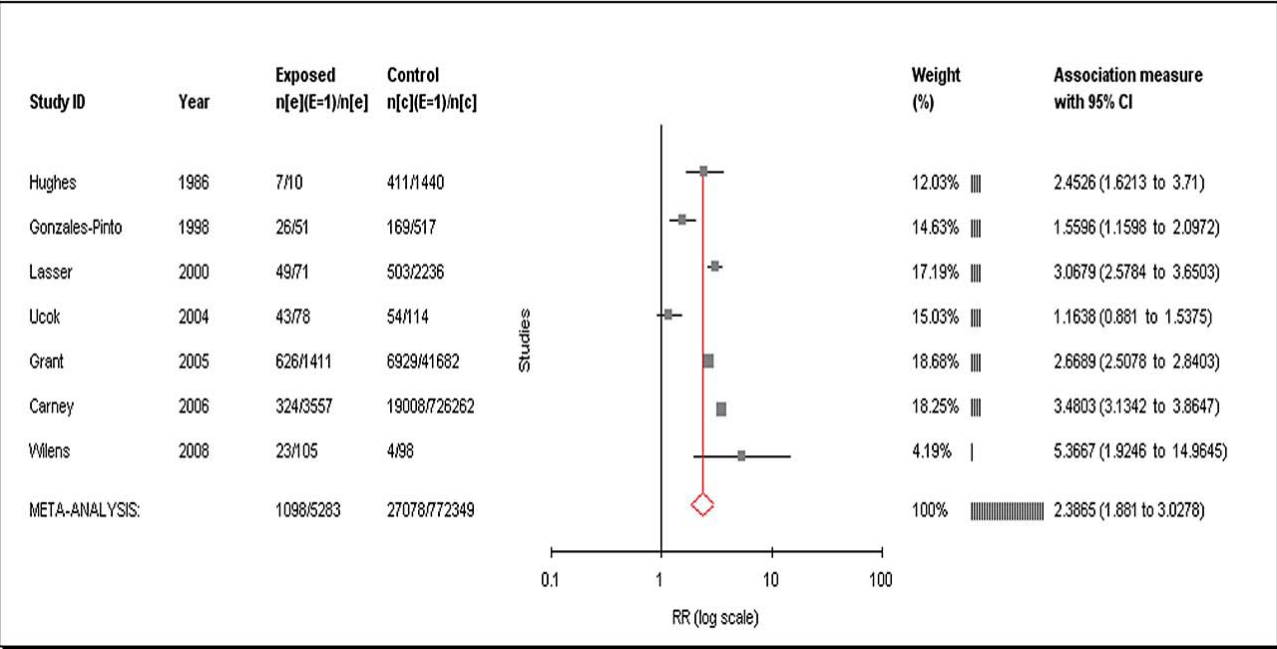
**Annotated forrest plot of relative risk for TUD among BD patients**. Study authors, dates, counts of smokers with BD, and counts of smokers among controls are shown on the left. On the right, the MIX software weights each study then calculates Relative Risk and 95% confidence intervals for TUD among BD patients. A graphical representation of this data is seen in the middle. The META-ANALYSIS study summarizes the weighted contributions of each individual study and shows a Relative Risk of 2.39 for TUD among BD patients, with a 95% confidence interval of 1.88 to 3.02.

Based on our fixed effects model, we estimated Relative Risk for TUD among BD patients at 2.77, with a p-value < 0.01, and a 95% confidence interval of 2.62 to 2.92. Based on the random effects model, we estimated Relative Risk for TUD among BD patients at 2.39, with a p-value < 0.0001, and 95% confidence interval of 1.88 to 3.03 (Figure 2). In the random effects model, Tau^2^, an estimate of between-study variance, is small and the Q-index, a measure of lack of credibility among the studies, is zero (Additional file 3, Table S1). Since the Relative Risk estimates are consistent across the two models, we proceed with the more conservative estimate of 2.39.

### Overlapping candidate genes

Gene2MeSH-nominated candidates for comorbid BD with TUD include: catechol-O-methyltransferase (COMT, Entrez GeneID 1312); solute carrier family 6 (neurotransmitter transporter, dopamine), member 3 (SLC6A3, GeneID 6531); solute carrier family 6 (neurotransmitter transporter, serotonin), member 4 (SLC6A4, GeneID 6532); tryptophan hydroxylase 1 (TPH1, GeneID 7166); and dopamine receptor D4 (DRD4, GeneID 1815). Validating these preliminary candidate genes by searching for at least two studies showing statistically significant positive association (Bonferroni corrected p-value ≤ 0.05) with both BD and TUD, we found that only COMT [42-47], SLC6A3 [48-57], and SLC6A4 [58-63] meet the requirement. TPH1 had one documented significant association with BD and one significant association with TUD, while DRD4 had four significant associations with TUD but only one significant association with BD.

### Common elements

**Figure 3:**
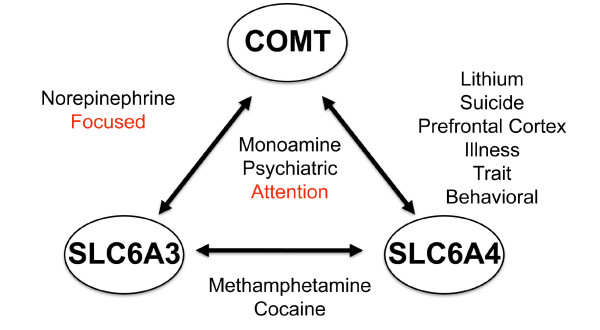
**PDG-ACE results**. Keywords are significantly over-represented in the Entrez Gene text at gene pairs (on the edges) or at all three genes (in the middle). These keywords pose hypotheses on the underlying biological associations between candidate genes and all are significantly over-represented (Bonferroni corrected p-value ≤ 0.05). In some cases, these keywords are expected, given the phenotype, while other keywords may reveal novel hypotheses on the underlying etiology associated with the phenotype.

For all three locus pairs formed by our overlapping candidate genes, PDG-ACE reports "monoamine, psychiatric, and attention" as significantly over-represented keywords (Figure 3). For the COMT/SLC6A4 pair, "lithium, suicide, prefrontal cortex, illness, trait, and behavioral" were significantly over-represented. For the COMT/SLC6A3 pair, "norepinephrine and focused" were significantly over-represented. For SLC6A3/SLC6A4, "methamphetamine and cocaine" were significantly over-represented. In assessing the context of these keywords in the Entrez Gene records of each locus, we noted that the keywords are consistent with psychiatric disorders, substance use disorders, and attention deficit hyperactivity disorder. In addition, the publications describing these effects indicate that these genes show gender specific effects with respect to both psychiatric disorders and substance use disorders [57,64-78]

GRAIL reported: "transporter, dopamine, serotonin, polymorphism, methyltransferase, genotype, allele, association, schizophrenia, disorder, dopaminergic, psychiatric, subjects, polymorphisms, uptake, attention, patients, anxiety, risk, and depression" as the keywords describing commonality among the overlapping candidate genes. As in the PDG-ACE analysis, we made note of the context of these keywords including: psychiatric disorders, neurotransmitter signaling, genetic variation, and attention. GRAIL quantifies similarity among loci (Table 1) and provides a single p-value to characterize the association. Note that ARVCF (Armadillo Repeat gene deletes in Velocardiofacial syndrome, GeneID 421) is adjacent to COMT on chromosome 22, so it is included in the GRAIL gene set.

**Table 1:** GRAIL output

GENE	GRAIL p-value	SELECTED SIMILAR GENES (Rank in parantheses)
COMT	0.001209104	ARVCF(5), SLC6A4(12), SLC6A3(25)

SLC6A3	0.001209104	SLC6A4(8), COMT(92)

SLC6A4	0.001209104	SLC6A3(16), COMT(97)

For each overlapping candidate gene, GRAIL quantifies the similarity to the other genes in the set (GRAIL p-value) and ranks genes by similarity. GRAIL also specifies keywords that characterize the similarity (see text).

### MiMI network

**Figure 4:**
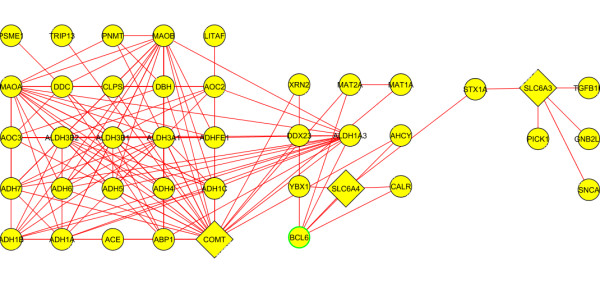
**MiMI network**. Overlapping candidates (COMT, SLC6A4, and SLC6A3) are triangular, while their interaction partners are circular.

The smallest network hypothesized by MiMI that contains all of the overlapping candidate genes has 41 genes total (Figure 4). We organized the graphic in three blocks, each anchored by one of the overlapping candidate genes. Table 2 displays the genes in the MiMI network. The MiMI database focuses primarily on protein-protein binding interactions, so edges in this network represent binding reactions between proteins coded by genes in the network. The genes in this network became input to the GAD analysis.

**Table 2:** Genes included in the MiMI network

Gene ID	Gene Name	Gene ID	Gene Name	Gene ID	Gene Name	Gene ID	Gene Name
26	ABP1	220	ALDH1A3	1644	DDC	6531	SLC6A3

1636	ACE	218	ALDH3A1	9416	DDX23	6532	SLC6A4

124	ADH1A	221	ALDH3B1	10399	GNB2L1	6622	SNCA

125	ADH1B	222	ALDH3B2	9516	LITAF	6804	STX1A

126	ADH1C	314	AOC2	4128	MAOA	7041	TGFB1I1

127	ADH4	8639	AOC3	4129	MAOB	9319	TRIP13

128	ADH5	604	BCL6	4143	MAT1A	22803	XRN2

130	ADH6	811	CALR	4144	MAT2A	4904	YBX1

131	ADH7	1208	CLPS	9463	PICK1		

137872	ADHFE1	1312	COMT	5409	PNMT		

191	AHCY	1621	DBH	5720	PSME1		

For each gene in the MiMI network, Entrez Gene ID and HGNC identifier are listed

### STRING network

**Figure 5:**
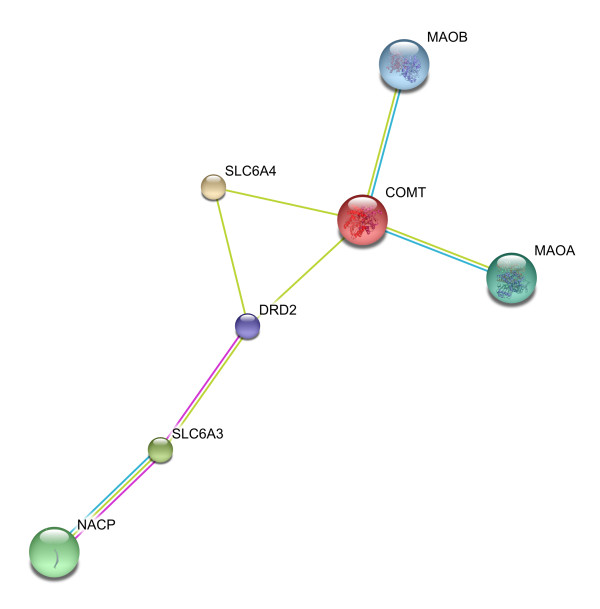
**STRING network**. The STRING network incorporates the overlapping candidates plus the genes that show the highest combined score characterizing the association among them.

The smallest network hypothesized by STRING, containing all of the overlapping candidate genes at the highest level of confidence is shown in Figure 5. To connect our overlapping candidates to each other, STRING added four nodes to the network. Genes in the resulting network include the overlapping candidates (COMT, SLC6A3, and SLC6A4) as well as SNCA [(synuclein, alpha (non A4 component of amyloid precursor), GeneID 6622), labeled as NACP in Figure 5]; DRD2 (dopamine receptor D2, GeneID 1813); MAOA (monoamine oxidase A, GeneID 4128); and MAOB (monoamine oxidase B, GeneID 4129). STRING reports multiple types of interactions and Table 3 reports the various association scores among the gene pairs. Note that we have omitted columns of zero scores from Table 3, though STRING also reports neighborhood score, fusion score, co-occurrence score, homology score, and co-expression score. STRING adds nodes based on decreasing "combined score", so DRD2 was the last node added, interacting with COMT, SLC6A3, and SLC6A4. The genes in this network became input to the GAD analysis.

**Table 3:** STRING genes and association scores

Node 1	Node 2	Experimental Score	Knowledge Score	Textmining Score	Combined Score
NACP	SLC6A3	0.873	0.9	0.481	0.993

MAOA	COMT	0	0.9	0.91	0.991

MAOB	COMT	0	0.9	0.848	0.984

DRD2	SLC6A3	0.644	0	0.955	0.983

DRD2	COMT	0	0	0.983	0.983

DRD2	SLC6A4	0	0	0.956	0.956

SLC6A4	COMT	0	0	0.933	0.933

Association between each gene pair is characterized by Experimental, Knowledge, and Textmining scores, as well as the Combined score. Columns of zero scores have been omitted.

### GeneGo networks

**Figure 6:**
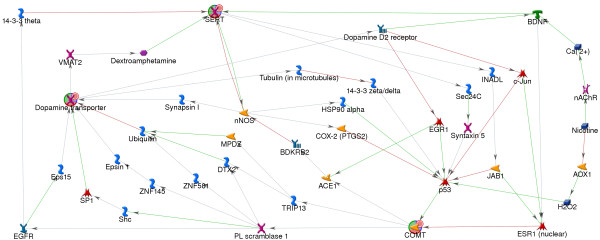
**GeneGo network, including nicotine**. The GeneGo network connects the overlapping candidates using the smallest number of nodes at the highest level of confidence for the edges. Nicotine and the nodes required to connect nicotine to the network have been added. Overlapping candidate genes (COMT, Dopamine transporter, and SERT) are shown as red, green, and blue circles. Other nodes are coded by the type of protein coded by the gene (e.g. kinases, transporters, etc.). Edges are labeled for direction of effect, where appropriate, and are green for activation or red for repression.

The smallest network hypothesized by GeneGo containing all three of the overlapping candidate genes required adding up to 4 nodes between each of the overlapping candidates, yielding a maximum path length of 5 edges, in a network containing 52 genes (Additional file 4: Figure S1). As with the MiMI and STRING networks, the genes in this network became input to the GAD analysis. This network, modified to include the 17 nodes that connect nicotine to the network (via p53, lower right, and BDNF, top right) is shown in Figure 6. The resulting network contains 69 genes, listed in Table 4, and the graphic is organized to illustrate the feedback loop formed by this network. GeneGo's built in pathways analysis did not reveal any significantly over-represented pathways. The 69 genes in this network were used in hypothesis testing via GAD, and subsequently were used in the ConceptGen analysis for differential gene expression.

**Table 4:** Genes included in the GeneGo network

Gene ID	Gene Name	Gene ID	Gene Name	Gene ID	Gene Name	Gene ID	Gene Name
1636	ACE	1145	CHRNE	5743	PTGS2	112714	TUBA3E

316	AOX1	1146	CHRNG	6233	RPS27A	7277	TUBA4A

624	BDKRB2	1312	COMT	9632	SEC24C	51807	TUBA8

627	BDNF	10987	COPS5	6464	SHC1	203068	TUBB

1134	CHRNA1	1813	DRD2	6571	SLC18A2	81027	TUBB1

57053	CHRNA10	113878	DTX2	6531	SLC6A3	7280	TUBB2A

1135	CHRNA2	1956	EGFR	6532	SLC6A4	347733	TUBB2B

1136	CHRNA3	1958	EGR1	6667	SP1	10383	TUBB2C

1137	CHRNA4	29924	EPN1	6811	STX5	10381	TUBB3

1138	CHRNA5	2060	EPS15	6853	SYN1	10382	TUBB4

8973	CHRNA6	2099	ESR1	7157	TP53	84617	TUBB6

1139	CHRNA7	3320	HSP90AA1	9319	TRIP13	7311	UBA52

55584	CHRNA9	10207	INADL	7846	TUBA1A	7314	UBB

1140	CHRNB1	3725	JUN	10376	TUBA1B	10971	YWHAQ

1141	CHRNB2	84708	LNX1	84790	TUBA1C	7534	YWHAZ

1142	CHRNB3	4842	NOS1	7278	TUBA3C	7704	ZBTB16

1143	CHRNB4	5359	PLSCR1	113457	TUBA3D	51545	ZNF581

1144	CHRND						

For each gene in the GeneGo network, including nicotine, Entrez Gene ID and HGNC identifier are listed

### Hypothesis testing: GAD

The MiMI network that includes the overlapping candidates (Additional file 3, Table S2) shows significant over-representation of genes associated with both BD and TUD but the network excluding the overlapping candidates (Additional file 3, Table S3) is not over-represented for either phenotype.

The STRING network including the overlapping candidates (Additional file 3, Table S4) is significantly associated with multiple measures of both BD and TUD. However, the STRING network that excludes the overlapping candidates (Additional file 3, Table S5) is over-represented only for "smoking behavior" (FDR 0.87%) and the more general term "mood disorder" (FDR 0.15%), rather than BD.

The original GeneGo network, including the overlapping candidates but excluding nicotine, is over-represented for genes associated with both BD and TUD (Additional file 3, Table S6). Excluding the overlapping candidates, this network is over represented only for the general term "depressive disorder, major" (Additional file 3, Table S7). After adding nicotine to this network, the GeneGo network containing 69 genes is over-represented for both BD and TUD associated genes, whether the overlapping candidates are included (Table 5) or excluded (Table 6). Excluding the overlapping candidates, this network is over-represented for genes associated with both "bipolar disorder" (FDR < 0.0001%) and "smoking behavior" (FDR = 0.0041%).

**Table 5:** GAD testing of the GeneGo network, including nicotine and the overlapping candidates

Term	Count	%	PValue	Genes	Fold Enrichment	FDR %
cognitive function	14	20.6	2.8E-18	1137, 6531, 6532, 1138, 1312, 1813, 57053, 1135, 2099, 627, 55584, 1139, 1141, 1142,	36.0	0.000

bipolar disorder	19	27.9	7.2E-15	1137, 6531, 1140, 1143, 8973, 6532, 1138, 1312, 1813, 1136, 57053, 1135, 1636, 627, 55584, 1134, 1139, 1141, 1142,	10.2	0.000

smoking behavior	10	14.7	1.9E-10	6531, 627, 55584, 6532, 4842, 1139, 1312, 1141, 1813, 1142,	21.9	0.000

Parkinson's disease	12	17.7	2.0E-07	1636, 1137, 6853, 2099, 6531, 627, 6532, 5743, 4842, 1312, 1813, 6571,	7.3	0.000

smoking	6	8.8	1.3E-05	1636, 6531, 7157, 6532, 1141, 1813,	18.1	0.025

methamphetamine abuse	5	7.4	2.1E-05	6531, 627, 6532, 1312, 1813,	27.3	0.042

alcoholism	7	10.3	2.2E-05	1636, 1137, 6531, 627, 6532, 1312, 1813,	11.3	0.042

Alzheimer's disease	13	19.1	5.3E-05	1137, 7157, 4842, 6532, 1312, 1136, 9632, 1636, 2099, 627, 5743, 1139, 1141,	3.8	0.104

attention deficit hyperactivity disorder	6	8.8	6.4E-05	1137, 6531, 627, 6532, 1312, 1813,	13.1	0.125

depression	6	8.8	7.2E-05	1636, 6531, 627, 6532, 1312, 1813,	12.8	0.141

depressive disorder, major	6	8.8	1.4E-04	2099, 627, 6532, 4842, 1139, 1813,	11.2	0.275

tardive dyskinesia	5	7.4	1.6E-04	6531, 6532, 4842, 1312, 1813,	16.8	0.318

personality traits	5	7.4	1.6E-04	6531, 627, 6532, 1312, 1813,	16.8	0.318

schizophrenia	13	19.1	3.3E-04	1137, 6531, 7157, 4842, 6532, 1312, 1813, 1636, 627, 5743, 1139, 1141, 6571,	3.1	0.645

mood pain	3	4.4	3.6E-04	627, 6532, 1312,	87.4	0.702

suicide	5	7.4	3.7E-04	1636, 6531, 6532, 4842, 1312,	13.7	0.729

obsessive compulsive disorder	4	5.9	5.3E-04	6531, 627, 6532, 1312,	23.3	1.027

Tourette syndrome	4	5.9	6.4E-04	6531, 6532, 1312, 1813,	21.9	1.253

eating disorders	3	4.4	7.1E-04	627, 6532, 1312,	65.6	1.390

alcoholism attention deficit hyperactivity disorder	3	4.4	7.1E-04	6532, 1312, 1813,	65.6	1.390

alcohol abuse smoking behavior	3	4.4	7.1E-04	6531, 6532, 1813,	65.6	1.390

mood disorder	4	5.9	7.7E-04	1636, 627, 6532, 1813,	20.6	1.508

heroin abuse	4	5.9	7.7E-04	6531, 6532, 1312, 1813,	20.6	1.508

anorexia nervosa	4	5.9	9.2E-04	2099, 627, 6532, 1312,	19.4	1.793

schizophrenia; tardive dyskinesia	3	4.4	1.2E-03	627, 6532, 1312,	52.5	2.290

dystonia, acute parkinsonism tardive dyskinesia	3	4.4	1.2E-03	6531, 6532, 1813,	52.5	2.290

neuroticism	3	4.4	1.2E-03	627, 6532, 1312,	52.5	2.290

obsessive-compulsive disorder	3	4.4	1.2E-03	627, 6532, 1312,	52.5	2.290

bipolar disorder schizophrenia	4	5.9	1.5E-03	627, 1139, 1312, 6571,	16.7	2.842

premenstrual dysphoric disorder	3	4.4	1.8E-03	2099, 6532, 1312,	43.7	3.393

kidney failure, chronic polycystic kidney disease	3	4.4	2.4E-03	624, 1636, 1956,	37.5	4.687

GAD testing, including nicotine and the overlapping candidates, shows significant over-representation for "bipolar disorder" (BD), "smoking behavior" (TUD), "smoking" (TUD), "alcohol abuse smoking behavior" (TUD) and "bipolar disorder, schizophrenia" (BD).

**Table 6:** GAD testing of the GeneGo network, including nicotine but excluding the overlapping candidates

Term	Count	%	PValue	Gene IDs	Fold Enrichment	FDR %
bipolar disorder	16	24.6	8.5E-12	1137, 1140, 1143, 8973, 1138, 1813, 1136, 57053, 1135, 1636, 627, 55584, 1134, 1139, 1141, 1142	9.34	0.0000

smoking behavior	7	10.8	2.1E-06	627, 55584, 4842, 1139, 1141, 1813, 1142	16.65	0.0041

GAD testing, including nicotine but excluding the overlapping candidates, shows significant over-representation for "bipolar disorder" (BD) and "smoking behavior" (TUD).

### Hypothesis testing: ConceptGen

ConceptGen finds that genes in the GeneGo network that includes nicotine are significantly over-represented (FDR 0.029) in one relevant GEO dataset, GSE10718 [79] (Table 7). GSE10718 is titled "Time course of NHBE cells exposed to whole cigarette smoke (full flavor)". Briefly, in this experiment, normal human bronchial epithelial cells were exposed to tobacco smoke for 15 minutes, and then incubated for 2 hours in fresh media. Gene expression was assayed on the Affymetrix HG-U133 plus 2 microarray, and differential expression was assessed by the ConceptGen expression analysis pipeline.

**Table 7:** ConceptGen results

Gene Symbol	Gene ID	p-value	Fold Chg
PTGS2	5743	5.0E-11	3.76

EGR1	1958	3.7E-07	2.45

JUN	3725	5.3E-06	1.96

UBB	7314	3.2E-09	1.62

HSP90AA1	3320	4.7E-05	1.23

TUBA4A	7277	5.7E-06	0.78

EPS15	2060	3.6E-05	0.74

TUBB	203068	7.1E-06	0.73

TUBB2C	10383	7.5E-07	0.72

ZNF581	51545	9.1E-05	0.71

TUBB6	84617	4.1E-07	0.70

BDKRB2	624	6.8E-05	0.69

TUBB3	10381	1.1E-07	0.59

13 Genes from the selected GeneGo network are differentially expressed with tobacco smoke exposure in GSE10718 (gene symbol and Entrez Gene ID, plus p-value and fold change calculated in the ConceptGen expression analysis pipeline).

### SNP prioritization via GIN

Based on genes in the GeneGo network that includes nicotine, for each SNP assayed in both NicSNP and GAIN, which also shows up in one of our candidate genes, we summed the GIN prioritization score and the transformed p-value [-log_10_(p-value)] from each of the NicSNP and GAIN studies. Additional file 5, Network_cand_SNPs_GIN_NicSNP_GAIN, provides SNPS prioritized for validation.

## Discussion

We found significant epidemiological evidence for increased risk of TUD among BD patients (Figure 2), as well as evidence of increased risk for BD among TUD patients, consistent with a common underlying etiology for these two disorders. There is clear evidence in the literature that both disorders are influenced by both genetic variation and the environment. Given evidence of common underlying etiology, as well as evidence of genetic and environmental influences on both BD and TUD, we hypothesized a common underlying *genetic *etiology, interacting with environmental nicotine exposure, influencing susceptibility to comorbid BD and TUD. We used multiple bioinformatics resources to test this hypothesis, including several novel resources.

### Meta-analysis

In the meta-analysis, we estimated relative risk for TUD among BD patients at approximately 2.39 times the risk for the general population. While we cannot discount the possibility of heterogeneity, testing suggests that these seven studies likely are representative of the same population. Equally, all seven studies show an increased Relative Risk for TUD among BD patients and, with the exception of Uock et al., the increased risk is statistically significant. We also cannot discount the possibility of publication bias in this analysis. However, the largest and most influential studies, Carney and Grant, were observed in large representative populations and are unlikely to have been influenced by this bias. MIX performed well on our data and we recommend its use for similar meta-analyses. These data are consistent with BD influencing TUD susceptibility, TUD influencing BD susceptibility, or both BD and TUD being influenced by some common element(s).

### Gene2MeSH

To identify candidate genes for the co-morbidity, we searched the extensive literature on both BD and TUD using Gene2MeSH [23]. Gene2MeSH nominates candidate genes that are significantly over-represented in literature annotated for the queried MeSH term. Gene2MeSH casts a very wide net (all of PubMed) in searching for gene/MeSH term pairs, performs the search in seconds, provides a Fisher Exact p-value to quantify the over-representation of each gene for a given MeSH term, and sets a threshold of Fisher Exact p-value ≤ 10^-4 ^to minimize spurious associations. While this p-value threshold is effective minimizing spurious associations, in the case of genes that have strong evidence of association in a relatively small body of literature, this threshold may exclude valid gene/MeSH term pairs. We used three queries for TUD ("tobacco use disorder", "smoking", and "nicotine"), though the query for "smoking" returned all three of the overlapping candidate genes. This suggests that, due to redundancy and ambiguity in MeSH annotation, the user must be wary in choosing the most appropriate MeSH terms for Gene2MeSH queries.

Gene2MeSH provides links that allow the user to follow the evidence for a gene/MeSH term pair by PubMed query. We used these links to validate our candidate genes by searching the literature for replicated, statistically significant (Bonferroni corrected), positive association with both BD and TUD. This combined threshold for over-representation and replication reduces the chance of a false positive association, though both false positives and false negatives remain possible. For example, both TPH1 and DRD4 show evidence of association with the comorbidity, but do not meet the criteria for replication. Future work may reveal these as true candidate genes for the comorbidity.

Notably, in this analysis and others that rely on published data, there is potential for bias in selecting well studied genes or well studied diseases in developing candidate genes. At the same time, analyses that do not depend on published disease association (e.g. interactions network analysis) may lack relevance to the disease phenotype of interest. We believe that by combining these approaches we minimize the effects of bias and maximize relevance to the phenotype of interest. Gene2MeSH performed well on our data and provided candidate genes for the analysis and we recommend its use for similar analyses. Alternately, other approaches to candidate gene selection (e.g. GWAS or microarray assay) are useful and could be substituted for Gene2MeSH in this analysis.

### Common elements

In an effort to understand how our overlapping candidates might interact in predisposing BD and TUD, we tested them for commonality using two relatively new algorithms: PDG-ACE [24,25] and GRAIL[26]. PDG-ACE finds significant commonality among these genes. Keywords such as "monoamine, psychiatric, norepinephrine, prefrontal cortex, illness, trait, and behavioral" are consistent with the expected roles of these genes in a psychiatric disorder. Keywords such as "lithium and suicide" are more specific to BD, while "methamphetamine and cocaine" are consistent with substance use disorders, common comorbidities for BD patients [80]. Keywords "focused and attention" are consistent with both Attention Deficit Disorder [81] and TUD [82]. The first two categories of keywords serve as positive controls, showing that PDG-ACE finds expected relationships among genes that were selected for association with BD and TUD. Note that in earlier testing of PDG-ACE, we used randomly selected locus pairs as negative controls to show that it does not find spurious associations [25]. Reviewing the publications associated with the keywords that PDG-ACE found significant, we found that these genes show gender specific effects in psychiatric and/or substance use disorders. This is consistent with gender differences seen in some studies of TUD susceptibility, though there are also genetic findings for TUD that have consistent effects in both genders [40,83-85]. BD susceptibility is often thought to be independent of gender, so the implication is that follow-on studies of comorbid BD with TUD might benefit from analyses that are stratified by gender or explicitly model gender in association testing. This benefit may extend to independent BD and TUD phenotypes for some genes.

GRAIL produced a set of keywords similar to those produced by PDG-ACE. Keywords "transporter, dopamine, serotonin, uptake, methyltransferase, dopaminergic, and psychiatric", are consistent with the functions of these genes in psychiatric disorders, while "subjects, polymorphisms, patients, risk, genotype, allele, and association" are consistent with the study of the genetic etiology of complex disease. Keywords "depression, anxiety, schizophrenia, disorder, and attention" are consistent with psychiatric disorders but do not highlight the comorbidities of BD with substance use. GRAIL could be improved by providing links to the references used in the analysis, which would allow the user to follow the keywords to assess their potential impact in the disease of interest and, potentially, recognize details such as the gender-specific effects of these genes in psychiatric and substance use disorders.

### Network hypothesis generation and testing

Hypothesis generation via MiMI, STRING, and GeneGo proceeded in parallel. MiMI focuses on protein-protein binding, while STRING and GeneGo also incorporate functional interactions based on the literature. The STRING network includes only 7 genes, the MiMI network includes 41 genes, the smaller GeneGo network includes 52 genes, and the GeneGo network with nicotine contains 69 genes. Testing via the Genetic Association Database reveals the MiMI, STRING, and smaller GeneGo networks as being generally consistent with the hypothesis. However, when excluding the overlapping candidates none of these networks provides statistically significant evidence in support of the hypothesis.

Only the GeneGo network that includes nicotine is statistically over-represented for genes associated with both BD and TUD. Interpreting these results, we believe that the GeneGo network that includes nicotine illustrates the importance of environmental nicotine exposure in both BD and TUD susceptibility. Consistent with this interpretation, the ConceptGen analysis of this network shows significant over-representation of genes that are differentially expressed with nicotine exposure. Notably, this network forms a feedback loop, where nicotine in the extracellular environment is sensed inside the cell via binding of nicotine to nicotinic acetylcholine receptors, influencing both calcium and neurotransmitter signaling. The environmental influence of nicotine would tend to be amplified over a number of cycles, potentially leading to a growing imbalance in the system with continued exposure. As such, we would expect neurotransmitter and/or calcium signaling to be increasingly imbalanced, over time, with exposure to nicotine. This result is also consistent our original hypothesized interaction between the genetic network and environmental nicotine exposure in predisposing both BD and TUD.

In these analyses, we use GAD for hypothesis testing. While GAD currently has 39,930 records, it remains under development, and it most certainly includes both false positive associations and is missing true associations. Another limitation is that GAD provides only dichotomous disease association data for each gene, which does not account for the strength of the evidence for association, sample size, and methodology. In spite of these limitations, GAD provides a means for obtaining quantitative measures of disease association for genetic networks and will become more valuable as more data are collected and vetted.

We note that the genes identified as being associated with BD and TUD in our GAD analysis are distinct from the genes found to be differentially expressed in the ConceptGen analysis. While this could happen if the data from GAD or ConceptGen represent false positive associations, we believe that this is a result of the differences in hypothesis testing. GAD looks for broad disease association, which could be caused by a range of diseases-related processes. Since we were focused on environmental effects on the network, we tested specifically for differential expression in the ConceptGen analysis. These are not mutually exclusive tests and may represent two views of the same or related phenomena.

While we emphasize that the selected network model is imperfect, it provides a reasonable model for the complex genetic and environmental influences on BD and TUD comorbidity. Interestingly, it also models other phenotypes. For instance, in the GAD analysis (Table 5) we see "kidney failure, chronic polycystic kidney disease", as a phenotype likely influenced by this network. Kidney failure is clearly a medical phenotype, rather than a psychiatric phenotype, but this network demonstrates genetic influences on the comorbidity and on this medical phenotype [86,87]. Consistent with this observation, many genes that are expressed in the brain are expressed in other tissues, so variation that influences psychiatric disorders may have whole body effects and influence what are traditionally considered medical disorders [88-90], thus blurring the boundaries between psychiatric and medical conditions.

### Common elements versus network analysis

In reviewing Table 5, the selected GeneGo network models multiple elements related to BD and TUD including: "cognitive function" [91,92], "Parkinson's disease" [93,94], multiple forms of substance abuse (alcohol, methamphetamine, heroin) [80,95], "attention deficit hyperactivity disorder" [81,82] (ADHD), and "premenstrual dysphoric disorder" [96,97]. Notably, these results are consistent with, and expand upon, the results seen in our PDG-ACE and GRAIL analyses. PDG-ACE keywords "monoamine, psychiatric, norepinephrine, prefrontal cortex, and behavioral", are consistent with network phenotypes "cognitive function" and "Parkinson's disease". PDG-ACE keywords "methamphetamine, and cocaine" are consistent with the network's associations with substance abuse, while PDG-ACE keywords "focused, and attention" are consistent with the network's ADHD association. GRAIL keywords "transporter, dopamine, serotonin, uptake, methyltransferase, and dopaminergic" are consistent with the network phenotypes "cognitive function" and "Parkinson's disease", while GRAIL keyword "attention" is consistent with the network phenotype ADHD. In addition, the gender specific effects we saw in following the PDG-ACE results are consistent with the network phenotype "premenstrual dysphoric disorder".

### Validation testing

Our initial efforts herein have prioritized 14,380 SNPs for validation based on the integration of evidence from GIN and GWAS. No specific biological inquiry has been implemented based on these findings yet, as this work represents a critical first step in planning further experiments. While this is a relatively large number of target SNPs, representing a correspondingly large number of hypothesis tests, the weights provided herein would also allow researchers to select a subset of these SNPs for validation. In addition, based on internal GeneGo annotation, this network proposes multiple potential drug targets for the comorbidity including: EGFR, SLC6A3, SLC6A4, Tubulin, DRD2, COX2, BDKRB2, ACE1, COMT, ESR1, and the NACHRs. Any or all of these genes provide ready targets for drug testing.

## Conclusions

The primary limitation of this approach relates to the validity of the published research in the literature and databases, which may be plagued by type I and II errors. In spite of this limitation, this research highlights several significant points. First, we hypothesized a common underlying *genetic *etiology, interacting with environmental nicotine exposure, influencing susceptibility for both BD and TUD. We find statistically significant evidence in support of this hypothesis in the selected GeneGo network, both via GAD testing for over-representation of BD and TUD associated genes, and ConceptGen testing for over-representation of differentially expressed genes. We see gender specific effects of our overlapping candidate genes, consistent with stratifying future BD and TUD analyses by gender or explicitly modeling gender in association analysis. Gene2MeSH provides a useful list of candidate genes for a particular phenotype (MeSH term), though it is not an exhaustive list. As seen in the selected GeneGo network, our overlapping Gene2MeSH candidates anchor the network and provide a means of identifying interactions that may be significant in disease, but other genes in the network are also viable candidates. Our definitions of psychiatric and medical conditions may be significantly modified as we progress in identifying genetic influences on complex diseases. Candidate genes and drug targets posed by this network may prove valuable in improving prognosis for patients with this comorbidity. In summary, our systems biology approach provides a model of interacting genetic influences, as well as gene-by-environment interactions, likely to impact comorbid DB with TUD.

## Abbreviations

BD: Bipolar Disorder; TUD: Tobacco Use Disorder; PDG-ACE: Prioritizing Disease Genes by Analysis of Common Elements; GRAIL: Gene Relationships Across Implicated Loci; GAD: Genetic Association Database; MiMI: Michigan Molecular Interactions database; STRING: Search Tool for the Retrieval of Interacting Genes/Proteins; COMT: catechol-O-methyltransferase; SLC6A3: solute carrier family 6 (neurotransmitter transporter, dopamine), member 3; SLC6A4: solute carrier family 6 (neurotransmitter transporter, serotonin), member 4; TPH1: tryptophan hydroxylase 1; DRD4: dopamine receptor D4.

## Competing interests

Financial: MGM has served as a consultant or on the speaker's bureau for Lilly, Pfizer, Merck, Astra-Zeneca, and Janssen Pharmaceuticals. SFS is listed as an inventor on a patent (US 20070258898) covering the use of certain SNPs in determining the diagnosis, prognosis, and treatment of addiction. NLS is the spouse of SFS and thus connected to the above patent. Non-financial: none.

## Authors' contributions

RCM conceived and initiated the study and was responsible for overall completion of the study. NLS and SFS provided expertise on nicotine and the NicSNP data. YDK, TK, and RKK provided research on drug effects, gender specific effects, and medical effects on the comorbidity, respectively. ASA and MAS provided expertise on Gene2MeSH and ConceptGen, respectively. JDC and MGM provided expert advice on study design and execution, and Bipolar Disorder. All authors read and approved the final manuscript.

## Pre-publication history

The pre-publication history for this paper can be accessed here:

http://www.biomedcentral.com/1471-2350/11/14/prepub

## Supplementary Material

Additional file 1 **Forest Notes**. An MS Excel spreadsheet showing the meta analysis input and output data as well as notes describing diagnostic criteria for BD and TUD, test and control population, and derivation of counts for each study. Click here for file

Additional file 2 **MOOSE Checklist**. An MS Word document that describes the details of the meta-analysis, consistent with the Reporting Checklist for Authors, Editors, and Reviewers of Meta-analyses of Observational Studies - Meta-analysis of Observational Studies in Epidemiology (MOOSE) criteria. Click here for file

Additional file 3 **Supplementary Tables**. An MS word document that provides the MIX meta-analysis summary report (Table S1), and 6 tables of GAD output (Tables S2 through S7). Click here for file

Additional file 4 **Figure S1**. A Portable Network Graphics file with the GeneGo network that connects the overlapping candidates using the smallest number of nodes at the highest level of confidence for the edges. Overlapping candidate genes (COMT, Dopamine transporter, and SERT) are shown as red, green, and blue circles. Other nodes are coded by the type of protein coded by the gene (e.g. kinases, transporters, etc.). Edges are labeled for direction of effect, where appropriate, and are green for activation or red for repression. Click here for file

Additional file 5 **Network_cand_SNPs_GIN_NicSNP_GAIN**. A comma separated text file of candidate SNPs prioritized for validation. Click here for file

## Aknowledgements

This work was supported by National Institutes of Health grant #U54 DA021519. SFS was supported by K01 DA024722.
