# Supplementary material for: Modeling complex genetic and environmental influences on comorbid bipolar disorder with tobacco use disorder
Source: BMC Med Genet. 2010 Jan 26;11:14. doi: 10.1186/1471-2350-11-14 (PMC2823619; doi:10.1186/1471-2350-11-14)
Supplement: Additional file 2 — MOOSE Checklist. An MS Word document that describes the details of the meta-analysis, consistent with the Reporting Checklist for Authors, Editors, and Reviewers of Meta-analyses of Observational Studies - Meta-analysis of Observational Studies in Epidemiology (MOOSE) criteria. [file 1471-2350-11-14-S2.DOCX]

Reporting Checklist for Authors, Editors, and Reviewers of Meta-analyses of Observational Studies (MOOSE criteria)

1. Problem definition
   1. Bipolar Disorder (BD) is a severe psychiatric disorder with a high rate of comorbid Tobacco Use Disorder (TUD). We seek to understand common underlying genetic and environmental influences on this comorbidity
2. Hypothesis statement
   1. We first hypothesized an increased risk for TUD among BD patients, relative to controls, and used meta-analysis of available data to test this hypothesis. (Follow-on analyses investigated genetic and environmental influences on this comorbidity.)
3. Description of study outcome(s)
   1. Relative Risk of TUD among BD patients and 95% Confidence Interval for each individual study and the meta-analysis
   2. Assessment of statistical significance of Relative Risk
4. Type of exposure or intervention used
   1. The exposed population includes individuals diagnosed with BD
   2. No intervention was used
5. Type of study designs used
   1. Observational studies
6. Study population
   1. Seven populations, see Supplemental Spreadsheet, Forest_notes.xls, and the original publications
7. Search strategy:
   1. Qualifications of searchers (eg, librarians and investigators)
      1. RCM (MS Biostatistics, PhD Human Genetics) conducted the search
   2. Search strategy, including time period included in the synthesis and keywords
      1. Search of all of PubMed (all available documents from all time periods)
      2. Query: “"Bipolar Disorder"[Mesh] AND ("Nicotine"[Mesh] OR "Tobacco"[Mesh] OR "Tobacco Use Disorder"[Mesh])”
   3. Effort to include all available studies, including contact with authors
      1. We followed the initial queries by searching the bibliographies of all papers returned from the PubMed query to identify data sources that may not have been included in the automated searches
      2. No contact was made with original authors
   4. Databases and registries searched
      1. PubMed
   5. Search software used, name and version, including special features used (eg, explosion)
      1. Queries were performed via an internet browser
   6. Use of hand searching (eg, reference lists of obtained articles)
      1. We followed the initial queries by searching the bibliographies of all papers returned from the PubMed query to identify data sources that may not have been included in the automated searches
   7. List of citations located and those excluded, including justification
      1. Manuscripts with appropriate counts of BD smokers and non-smokers, control smokers and non-smokers, or manuscripts with data sufficient to derive the necessary counts were used
      2. All other manuscripts were excluded
   8. Method of addressing articles published in languages other than English
      1. All references returned were in English or had English translations
   9. Method of handling abstracts and unpublished studies
      1. All manuscripts were available by direct download via the University of Michigan library or by interlibrary loan
      2. Unpublished data was not used
   10. Description of any contact with authors
       1. None
8. Reporting of methods should include:
   1. Description of relevance or appropriateness of studies assembled for assessing the hypothesis to be tested
      1. Studies were selected for relevance and appropriateness of the data in testing the hypothesis
      2. Manuscripts with appropriate counts of BD smokers and non-smokers, control smokers and non-smokers, or manuscripts with data sufficient to derive the necessary counts were used
   2. Rationale for the selection and coding of data (eg, sound clinical principles or convenience)
      1. Coding consisted simply of counts of BD smokers and non-smokers, control smokers and non-smokers
   3. Documentation of how data were classified and coded (eg, multiple raters, blinding, and inter-rater reliability)
      1. See Supplemental Spreadsheet, Forest_notes.xls and the original publications for data on individual studies
   4. Assessment of confounding (eg, comparability of cases and controls in studies where appropriate)
      1. See supplemental Spreadsheet, Forest_notes.xls and the original publications for control criteria
   5. Assessment of study quality, including blinding of quality assessors; stratification or regression on possible predictors of study results
      1. See supplemental Spreadsheet, Forest_notes.xls and the original publications for data on individual studies
   6. Assessment of heterogeneity
      1. Heterogeneity was assessed by Cochran’s Q, Higgin’s H, and Higgin’s I^2^ statisitcs, see Supplemental Table 5
   7. Description of statistical methods (eg, complete description of fixed or random effects models, justification of whether the chosen models account for predictors of study results, dose-response models, or cumulative meta-analysis) in sufficient detail to be replicated
      1. Using MIX software and the data in Supplemental Spreadsheet, Forest_notes.xls, we generated an annotated forest plot of Relative Risk, modeled smoking as a random effect, and used DerSimonian-Laird weighting when combining data from the individual studies. All other settings are MIX defaults, found under the Analysis/preferences tab.
   8. Provision of appropriate tables and graphics
      1. See Supplemental Table 5, Forest_Notes.xls, and Figure 2
9. Reporting of results should include:
   1. Graphic summarizing individual study estimates and overall estimate
      1. See Figure 2
   2. Table giving descriptive information for each study included
      1. See Forest _Notes.xls
   3. Results of sensitivity testing (eg, subgroup analysis)
      1. None, due to the small set of observations
   4. Indication of statistical uncertainty of findings
      1. See Figure 2 and Supplemental Table 5
10. Reporting of discussion should include:
    1. Quantitative assessment of bias (eg, publication bias)
       1. See Results
    2. Justification for exclusion (eg, exclusion of non–English-language citations)
       1. All studies were available in English. All studies with appropriate counts were included.
    3. Assessment of quality of included studies
       1. See Results
11. Reporting of conclusions should include:
    1. Consideration of alternative explanations for observed results
       1. See Results
    2. Generalization of the conclusions (ie, appropriate for the data presented and within the domain of the literature review)
       1. See Results
    3. Guidelines for future research
       1. Results of this meta-analysis became input to follow-on analyses, examining genetic and environmental influences on the comorbidity.
    4. Disclosure of funding source
       1. See Acknowledgements
