# Supplementary material for: Modeling complex genetic and environmental influences on comorbid bipolar disorder with tobacco use disorder
Source: BMC Med Genet. 2010 Jan 26;11:14. doi: 10.1186/1471-2350-11-14 (PMC2823619; doi:10.1186/1471-2350-11-14)
Supplement: Additional file 3 — Supplementary Tables. An MS word document that provides the MIX meta-analysis summary report (Table S1), and 6 tables of GAD output (Tables S2 through S7). [file 1471-2350-11-14-S3.DOC]

# Supplementary tables

### Table S1 -MIX Meta-Analysis Summary Report

| **Number of studies** |  | 7 |  |
| --- | --- | --- | --- |
| **Number of participants** |  | 777,632 |  |
| **RR (DL) - Random effects model** |  |  |  |
| **Meta-analysis outcome** |  | 2.3865 |  |
| 95% CI lower limit |  | 1.881 |  |
| 95% CI upper limit |  | 3.0278 |  |
| **z** |  | 7.1631 |  |
| p-value (two-tailed) |  | < 0.0001 |  |
| **Heterogeneity** |  |  |  |
| t^2 |  | 0.0779 |  |
| Q-index |  | 0 |  |

The meta-analysis included 7 studies, involving 777,632 study subjects and assessing risk of comorbid TUD among BD patients, relative to the general population. The Random Effects model with DerSimonian Laird weighting estimates Relative Risk at 2.39, with a p-value < 0.0001. Heterogeneity between studies is not significant based on Tau^2^, an estimate of between study variance, and Q-index, a measure of lack of credibility among the studies.

### Table S2 - GAD testing of the MiMI network (including overlapping candidates)

| **Term** | **Count** | **%** | **PValue** | **Genes** | **Fold Enrichment** | **FDR %** |
| --- | --- | --- | --- | --- | --- | --- |
| attention deficit hyperactivity disorder | 7 | 18.0 | 2.8E-07 | 5409, 4129, 6531, 6532, 1312, 4128, 1621, | 22.65 | 0.001 |
| Parkinson's disease | 10 | 25.6 | 3.7E-07 | 1636, 4129, 6531, 6532, 1312, 4128, 6622, 124, 125, 126, 1621, 127, | 9.05 | 0.001 |
| alcohol abuse | 7 | 18.0 | 6.6E-07 | 6531, 6532, 1312, 4128, 6622, 124, 125, 126, 127, | 19.69 | 0.001 |
| drug dependence | 4 | 10.3 | 3.6E-06 | 128, 131, 130, 124, 125, 126, | 103.52 | 0.007 |
| Tourette syndrome | 5 | 12.8 | 4.0E-06 | 6531, 6532, 1312, 4128, 1621, | 40.44 | 0.008 |
| smoking behavior | 6 | 15.4 | 8.0E-06 | 4129, 6531, 6532, 1312, 4128, 1621, | 19.41 | 0.016 |
| body mass obesity | 4 | 10.3 | 2.9E-05 | 4129, 6531, 1312, 4128, | 57.51 | 0.057 |
| tardive dyskinesia | 5 | 12.8 | 3.1E-05 | 4129, 6531, 6532, 1312, 4128, | 24.88 | 0.061 |
| personality traits | 5 | 12.8 | 3.1E-05 | 6531, 6532, 1312, 4128, 127, | 24.88 | 0.061 |
| alcoholism | 6 | 15.4 | 3.6E-05 | 1636, 6531, 6532, 1312, 4128, 124, 125, 126, | 14.38 | 0.070 |
| attention deficit disorder conduct disorder oppositional defiant disorder | 6 | 15.4 | 5.5E-05 | 5409, 6531, 6532, 1312, 4128, 1621, | 13.16 | 0.109 |
| alcohol dependence | 5 | 12.8 | 7.3E-05 | 128, 6532, 131, 130, 124, 125, 126, | 20.22 | 0.143 |
| suicide | 5 | 12.8 | 7.3E-05 | 1636, 6531, 6532, 1312, 4128, | 20.22 | 0.143 |
| cognitive function | 5 | 12.8 | 9.3E-05 | 6531, 6532, 1312, 4128, 6622, | 19.03 | 0.183 |
| ADHD | 4 | 10.3 | 9.8E-05 | 6532, 1312, 4128, 1644, | 39.82 | 0.192 |
| schizophrenia | 11 | 28.2 | 1.3E-04 | 9463, 1636, 4129, 6531, 218, 221, 6532, 1312, 4128, 1644, 1621, | 3.93 | 0.247 |
| obsessive compulsive disorder | 4 | 10.3 | 1.5E-04 | 6531, 6532, 1312, 4128, | 34.51 | 0.302 |
| attention deficit disorder | 3 | 7.7 | 1.6E-04 | 6531, 6532, 4128, | 129.40 | 0.309 |
| depressive disorder, major endocrine regulation | 3 | 7.7 | 1.6E-04 | 6532, 1312, 4128, | 129.40 | 0.309 |
| depression | 5 | 12.8 | 2.0E-04 | 1636, 6531, 6532, 1312, 4128, | 15.78 | 0.385 |
| mood disorder | 4 | 10.3 | 2.3E-04 | 1636, 4129, 6532, 4128, | 30.45 | 0.447 |
| shyness | 3 | 7.7 | 3.1E-04 | 6532, 1312, 4128, | 97.05 | 0.614 |
| headache | 3 | 7.7 | 3.1E-04 | 6531, 1312, 4128, | 97.05 | 0.614 |
| pain response | 3 | 7.7 | 5.2E-04 | 6532, 1312, 4128, | 77.64 | 1.017 |
| neuroticism | 3 | 7.7 | 5.2E-04 | 6532, 1312, 4128, | 77.64 | 1.017 |
| post-traumatic stress disorder | 3 | 7.7 | 5.2E-04 | 4129, 6531, 6532, | 77.64 | 1.017 |
| obsessive-compulsive disorder | 3 | 7.7 | 5.2E-04 | 6532, 1312, 4128, | 77.64 | 1.017 |
| premenstrual dysphoric disorder | 3 | 7.7 | 7.8E-04 | 6532, 1312, 4128, | 64.70 | 1.515 |
| BP-Major Depressive | 3 | 7.7 | 7.8E-04 | 6532, 1312, 4128, | 64.70 | 1.515 |
| Manic-depressive illness | 3 | 7.7 | 1.1E-03 | 6531, 6532, 4128, | 55.46 | 2.106 |
| migraine; migraine with aura | 3 | 7.7 | 1.4E-03 | 4129, 6532, 4128, | 48.53 | 2.786 |
| bipolar disorder; depression | 3 | 7.7 | 1.4E-03 | 1636, 6532, 4128, | 48.53 | 2.786 |
| migraine | 4 | 10.3 | 2.6E-03 | 1636, 6532, 4128, 1621, | 13.62 | 4.897 |

GAD testing, including the overlapping candidates, shows significant over-representation for "smoking behavior" (TUD), "Manic Depressive Illness" (BD) "Bipolar Disorder, Depression" (BD) and the more general but related phenotype "Mood Disorder" (highlighted in bold italics).

## Table S3 - GAD testing of the MiMI network (excluding overlapping candidates)

| **Term** | **Count** | **%** | **PValue** | **Gene IDs** | **Fold Enrichment** | **FDR %** |
| --- | --- | --- | --- | --- | --- | --- |
| drug dependence | 4 | 11.1 | 2.3E-06 | 128, 131, 130, 124, 125, 126, | 117.64 | 0.00 |
| Parkinson's disease | 7 | 19.4 | 2.1E-04 | 1636, 4129, 4128, 6622, 124, 125, 126, 1621, 127, | 7.20 | 0.41 |
| alcohol dependence | 4 | 11.1 | 1.0E-03 | 128, 131, 130, 124, 125, 126, | 18.38 | 2.02 |
| attention deficit hyperactivity disorder | 4 | 11.1 | 2.0E-03 | 5409, 4129, 4128, 1621, | 14.70 | 3.85 |

GAD testing, excluding the overlapping candidates, shows no over-representation for BD or TUD phenotypes

## Table S4 - GAD testing of the STRING network (including overlapping candidates)

| **Term** | **Count** | **%** | **PValue** | **Genes** | **Fold Enrichment** | **FDR %** |
| --- | --- | --- | --- | --- | --- | --- |
| tardive dyskinesia | 6 | 85.7 | 1.3E-10 | 4129, 6531, 6532, 1312, 1813, 4128, | 106.6 | 0.0000 |
| cognitive function | 6 | 85.7 | 5.6E-10 | 6531, 6532, 1312, 1813, 4128, 6622, | 81.6 | 0.0000 |
| attention deficit hyperactivity disorder | 6 | 85.7 | 1.3E-09 | 4129, 6531, 6532, 1312, 1813, 4128, | 69.3 | 0.0000 |
| smoking behavior | 6 | 85.7 | 1.3E-09 | 4129, 6531, 6532, 1312, 1813, 4128, | 69.3 | 0.0000 |
| alcohol abuse | 6 | 85.7 | 2.8E-09 | 6531, 6532, 1312, 1813, 4128, 6622, | 60.3 | 0.0000 |
| Tourette syndrome | 5 | 71.4 | 6.0E-09 | 6531, 6532, 1312, 1813, 4128, | 144.4 | 0.0000 |
| Parkinson's disease | 7 | 100.0 | 6.7E-09 | 4129, 6531, 6532, 1312, 1813, 4128, 6622, | 22.6 | 0.0000 |
| personality traits | 5 | 71.4 | 4.9E-08 | 6531, 6532, 1312, 1813, 4128, | 88.9 | 0.0001 |
| body mass obesity | 4 | 57.1 | 3.0E-07 | 4129, 6531, 1312, 4128, | 205.4 | 0.0006 |
| depression | 5 | 71.4 | 3.3E-07 | 6531, 6532, 1312, 1813, 4128, | 56.4 | 0.0006 |
| alcoholism | 5 | 71.4 | 1.0E-06 | 6531, 6532, 1312, 1813, 4128, | 42.8 | 0.0020 |
| attention deficit disorder conduct disorder oppositional defiant disorder | 5 | 71.4 | 1.5E-06 | 6531, 6532, 1312, 1813, 4128, | 39.2 | 0.0029 |
| obsessive compulsive disorder | 4 | 57.1 | 1.6E-06 | 6531, 6532, 1312, 4128, | 123.2 | 0.0031 |
| methamphetamine abuse | 4 | 57.1 | 2.0E-06 | 6531, 6532, 1312, 1813, | 115.5 | 0.0039 |
| mood disorder | 4 | 57.1 | 2.4E-06 | 4129, 6532, 1813, 4128, | 108.7 | 0.0047 |
| heroin abuse | 4 | 57.1 | 2.4E-06 | 6531, 6532, 1312, 1813, | 108.7 | 0.0047 |
| mood disorders | 4 | 57.1 | 2.4E-06 | 6532, 1312, 1813, 4128, | 108.7 | 0.0047 |
| attention deficit disorder | 3 | 42.9 | 8.6E-06 | 6531, 6532, 4128, | 462.1 | 0.0169 |
| depressive disorder, major endocrine regulation | 3 | 42.9 | 8.6E-06 | 6532, 1312, 4128, | 462.1 | 0.0169 |
| alcoholism attention deficit hyperactivity disorder | 3 | 42.9 | 1.7E-05 | 6532, 1312, 1813, | 346.6 | 0.0337 |
| shyness | 3 | 42.9 | 1.7E-05 | 6532, 1312, 4128, | 346.6 | 0.0337 |
| alcohol abuse smoking behavior | 3 | 42.9 | 1.7E-05 | 6531, 6532, 1813, | 346.6 | 0.0337 |
| headache | 3 | 42.9 | 1.7E-05 | 6531, 1312, 4128, | 346.6 | 0.0337 |
| suicide | 4 | 57.1 | 1.7E-05 | 6531, 6532, 1312, 4128, | 57.8 | 0.0339 |
| antisocial personality disorder | 3 | 42.9 | 2.9E-05 | 6531, 1813, 4128, | 277.3 | 0.0562 |
| pain response | 3 | 42.9 | 2.9E-05 | 6532, 1312, 4128, | 277.3 | 0.0562 |
| bipolar disorder; major depressive disorder; rapid cycling mood disorder | 3 | 42.9 | 2.9E-05 | 1312, 1813, 4128, | 277.3 | 0.0562 |
| dystonia, acute parkinsonism tardive dyskinesia | 3 | 42.9 | 2.9E-05 | 6531, 6532, 1813, | 277.3 | 0.0562 |
| neuroticism | 3 | 42.9 | 2.9E-05 | 6532, 1312, 4128, | 277.3 | 0.0562 |
| post-traumatic stress disorder | 3 | 42.9 | 2.9E-05 | 4129, 6531, 6532, | 277.3 | 0.0562 |
| obsessive-compulsive disorder | 3 | 42.9 | 2.9E-05 | 6532, 1312, 4128, | 277.3 | 0.0562 |
| premenstrual dysphoric disorder | 3 | 42.9 | 4.3E-05 | 6532, 1312, 4128, | 231.1 | 0.0842 |
| BP-Major Depressive | 3 | 42.9 | 4.3E-05 | 6532, 1312, 4128, | 231.1 | 0.0842 |
| Manic-depressive illness | 3 | 42.9 | 6.0E-05 | 6531, 6532, 4128, | 198.1 | 0.1177 |
| cocaine abuse | 3 | 42.9 | 8.0E-05 | 6531, 6532, 1813, | 173.3 | 0.1568 |
| migraine; migraine with aura | 3 | 42.9 | 8.0E-05 | 4129, 6532, 4128, | 173.3 | 0.1568 |
| bipolar disorder; depression | 3 | 42.9 | 8.0E-05 | 6532, 1813, 4128, | 173.3 | 0.1568 |
| cirrhosis, alcoholic; alcoholism | 3 | 42.9 | 8.0E-05 | 6531, 6532, 1813, | 173.3 | 0.1568 |
| bipolar disorder | 5 | 71.4 | 8.6E-05 | 6531, 6532, 1312, 1813, 4128, | 14.2 | 0.1690 |
| schizophrenia | 6 | 85.7 | 9.3E-05 | 4129, 6531, 6532, 1312, 1813, 4128, | 7.7 | 0.1830 |
| substance abuse | 3 | 42.9 | 1.0E-04 | 6532, 1312, 1813, | 154.0 | 0.2014 |
| chronic fatigue syndrome | 4 | 57.1 | 1.6E-04 | 4129, 6532, 1312, 4128, | 27.6 | 0.3186 |
| anxiety disorder | 3 | 42.9 | 2.2E-04 | 6532, 1312, 4128, | 106.6 | 0.4343 |
| personality disorders | 3 | 42.9 | 2.2E-04 | 6532, 1813, 4128, | 106.6 | 0.4343 |
| ADHD | 3 | 42.9 | 2.2E-04 | 6532, 1312, 4128, | 106.6 | 0.4343 |
| weight gain | 3 | 42.9 | 4.3E-04 | 6531, 1312, 1813, | 77.0 | 0.8468 |
| smoking | 3 | 42.9 | 1.1E-03 | 6531, 6532, 1813, | 47.8 | 2.2122 |
| panic disorder | 3 | 42.9 | 1.3E-03 | 6532, 1312, 4128, | 44.7 | 2.5256 |
| migraine | 3 | 42.9 | 2.0E-03 | 6532, 1813, 4128, | 36.5 | 3.7732 |

GAD testing, including the overlapping candidates, shows significant over-representation for multiple BD and TUD related phenotypes.

## Table S5 - STRING Network (excluding overlapping candidates)

| **Term** | **Count** | **%** | **PValue** | **Genes** | **Fold Enrichment** | **FDR %** |
| --- | --- | --- | --- | --- | --- | --- |
| mood disorder | 3 | 75.0 | 7.8E-05 | 4129, 1813, 4128, | 142.7 | 0.15 |
| Parkinson's disease | 4 | 100.0 | 8.5E-05 | 4129, 1813, 4128, 6622, | 22.6 | 0.17 |
| tardive dyskinesia | 3 | 75.0 | 1.9E-04 | 4129, 1813, 4128, | 93.3 | 0.36 |
| cognitive function | 3 | 75.0 | 3.2E-04 | 1813, 4128, 6622, | 71.4 | 0.63 |
| smoking behavior | 3 | 75.0 | 4.4E-04 | 4129, 1813, 4128, | 60.7 | 0.87 |
| attention deficit hyperactivity disorder | 3 | 75.0 | 4.4E-04 | 4129, 1813, 4128, | 60.7 | 0.87 |
| alcohol abuse | 3 | 75.0 | 5.9E-04 | 1813, 4128, 6622, | 52.7 | 1.15 |

GAD testing, excluding the overlapping candidates, shows over-representation only for "smoking behavior" and the general term "mood disorder" (highlighted in bold italics).

**Table S6 - GAD testing of the GeneGo network (including overlapping candidates but excluding nicotine)**

| **Term** | **Count** | **%** | **PValue** | **Genes** | **Fold Enrichment** | **FDR %** |
| --- | --- | --- | --- | --- | --- | --- |
| Parkinson's disease | 11 | 21.6 | 4.85E-09 | 1636, 6853, 2099, 6531, 627, 6532, 5743, 4842, 1312, 1813, 6571, | 11.31 | 0.00 |
| cognitive function | 6 | 11.8 | 1.71E-06 | 2099, 6531, 627, 6532, 1312, 1813, | 25.95 | 0.00 |
| methamphetamine abuse | 5 | 9.8 | 2.27E-06 | 6531, 627, 6532, 1312, 1813, | 45.95 | 0.00 |
| smoking behavior | 6 | 11.8 | 3.93E-06 | 6531, 627, 6532, 4842, 1312, 1813, | 22.06 | 0.01 |
| depression | 6 | 11.8 | 4.46E-06 | 1636, 6531, 627, 6532, 1312, 1813, | 21.52 | 0.01 |
| alcoholism | 6 | 11.8 | 1.78E-05 | 1636, 6531, 627, 6532, 1312, 1813, | 16.34 | 0.04 |
| personality traits | 5 | 9.8 | 1.79E-05 | 6531, 627, 6532, 1312, 1813, | 28.28 | 0.04 |
| tardive dyskinesia | 5 | 9.8 | 1.79E-05 | 6531, 6532, 4842, 1312, 1813, | 28.28 | 0.04 |
| smoking | 5 | 9.8 | 2.81E-05 | 1636, 6531, 7157, 6532, 1813, | 25.35 | 0.06 |
| suicide | 5 | 9.8 | 4.20E-05 | 1636, 6531, 6532, 4842, 1312, | 22.98 | 0.08 |
| obsessive compulsive disorder | 4 | 7.8 | 1.02E-04 | 6531, 627, 6532, 1312, | 39.21 | 0.20 |
| attention deficit hyperactivity disorder | 5 | 9.8 | 1.03E-04 | 6531, 627, 6532, 1312, 1813, | 18.38 | 0.20 |
| mood pain | 3 | 5.9 | 1.20E-04 | 627, 6532, 1312, | 147.05 | 0.24 |
| Tourette syndrome | 4 | 7.8 | 1.25E-04 | 6531, 6532, 1312, 1813, | 36.76 | 0.25 |
| mood disorder | 4 | 7.8 | 1.51E-04 | 1636, 627, 6532, 1813, | 34.60 | 0.30 |
| heroin abuse | 4 | 7.8 | 1.51E-04 | 6531, 6532, 1312, 1813, | 34.60 | 0.30 |
| anorexia nervosa | 4 | 7.8 | 1.81E-04 | 2099, 627, 6532, 1312, | 32.68 | 0.35 |
| depressive disorder, major | 5 | 9.8 | 1.95E-04 | 2099, 627, 6532, 4842, 1813, | 15.64 | 0.38 |
| schizophrenia | 10 | 19.6 | 2.15E-04 | 1636, 6531, 627, 7157, 6532, 5743, 4842, 1312, 1813, 6571, | 4.06 | 0.42 |
| alcoholism attention deficit hyperactivity disorder | 3 | 5.9 | 2.39E-04 | 6532, 1312, 1813, | 110.28 | 0.47 |
| alcohol abuse smoking behavior | 3 | 5.9 | 2.39E-04 | 6531, 6532, 1813, | 110.28 | 0.47 |
| eating disorders | 3 | 5.9 | 2.39E-04 | 627, 6532, 1312, | 110.28 | 0.47 |
| Alzheimer's disease | 9 | 17.7 | 3.46E-04 | 9632, 1636, 2099, 627, 7157, 6532, 5743, 4842, 1312, | 4.41 | 0.68 |
| dystonia, acute parkinsonism tardive dyskinesia | 3 | 5.9 | 3.97E-04 | 6531, 6532, 1813, | 88.23 | 0.78 |
| neuroticism | 3 | 5.9 | 3.97E-04 | 627, 6532, 1312, | 88.23 | 0.78 |
| schizophrenia; tardive dyskinesia | 3 | 5.9 | 3.97E-04 | 627, 6532, 1312, | 88.23 | 0.78 |
| obsessive-compulsive disorder | 3 | 5.9 | 3.97E-04 | 627, 6532, 1312, | 88.23 | 0.78 |
| cardiovascular disease | 5 | 9.8 | 4.74E-04 | 1636, 2099, 7157, 5743, 81027, | 12.46 | 0.93 |
| attention deficit disorder conduct disorder oppositional defiant disorder | 5 | 9.8 | 4.74E-04 | 2099, 6531, 6532, 1312, 1813, | 12.46 | 0.93 |
| Myocardial Infarction | 5 | 9.8 | 5.74E-04 | 624, 1636, 2099, 6532, 5743, | 11.86 | 1.12 |
| premenstrual dysphoric disorder | 3 | 5.9 | 5.93E-04 | 2099, 6532, 1312, | 73.52 | 1.16 |
| prostate cancer | 7 | 13.7 | 7.57E-04 | 1636, 2099, 7157, 5743, 1956, 1312, 10381, | 5.69 | 1.48 |
| kidney failure, chronic polycystic kidney disease | 3 | 5.9 | 8.27E-04 | 624, 1636, 1956, | 63.02 | 1.61 |
| breast cancer | 9 | 17.7 | 9.72E-04 | 1636, 2099, 7157, 203068, 5743, 1956, 1312, 6464, 1813, | 3.79 | 1.89 |
| cocaine abuse | 3 | 5.9 | 1.10E-03 | 6531, 6532, 1813, | 55.14 | 2.13 |
| cirrhosis, alcoholic; alcoholism | 3 | 5.9 | 1.10E-03 | 6531, 6532, 1813, | 55.14 | 2.13 |
| bipolar disorder; depression | 3 | 5.9 | 1.10E-03 | 1636, 6532, 1813, | 55.14 | 2.13 |
| obesity | 6 | 11.8 | 1.13E-03 | 624, 1636, 2099, 6531, 6532, 10381, | 6.84 | 2.20 |
| substance abuse | 3 | 5.9 | 1.41E-03 | 6532, 1312, 1813, | 49.02 | 2.73 |
| migraine | 4 | 7.8 | 1.72E-03 | 1636, 2099, 6532, 1813, | 15.48 | 3.32 |

GAD testing, including the overlapping candidates, shows significant over-representation for multiple BD and TUD related phenotypes (highlighted in bold italics).

## Table S7 - GeneGo Network (excluding both overlapping candidates and nicotine)

| **Term** | **Count** | **%** | **PValue** | **Genes** | **Fold Enrichment** | **FDR %** |
| --- | --- | --- | --- | --- | --- | --- |
| Parkinson's disease | 8 | 16.7 | 6.0E-06 | 1636, 6853, 2099, 627, 5743, 4842, 1813, 6571, | 9.5 | 0.01 |
| cardiovascular disease | 5 | 10.4 | 2.5E-04 | 1636, 2099, 7157, 5743, 81027, | 14.4 | 0.49 |
| kidney failure, chronic polycystic kidney disease | 3 | 6.3 | 6.0E-04 | 624, 1636, 1956, | 73.0 | 1.18 |
| breast cancer | 8 | 16.7 | 1.8E-03 | 1636, 2099, 7157, 203068, 5743, 1956, 1813, 6464, | 3.9 | 3.42 |
| depressive disorder, major | 4 | 8.3 | 2.0E-03 | 2099, 627, 4842, 1813, | 14.5 | 3.88 |
| prostate cancer | 6 | 12.5 | 2.4E-03 | 1636, 2099, 7157, 5743, 1956, 10381, | 5.6 | 4.70 |

GAD testing, excluding the overlapping candidates and nicotine, shows over-representation only for the general term "depressive disorder, major" (highlighted in bold italics).
