# Supplementary figures and images for: Modeling complex genetic and environmental influences on comorbid bipolar disorder with tobacco use disorder
Source: BMC Med Genet. 2010 Jan 26;11:14. doi: 10.1186/1471-2350-11-14 (PMC2823619; doi:10.1186/1471-2350-11-14)

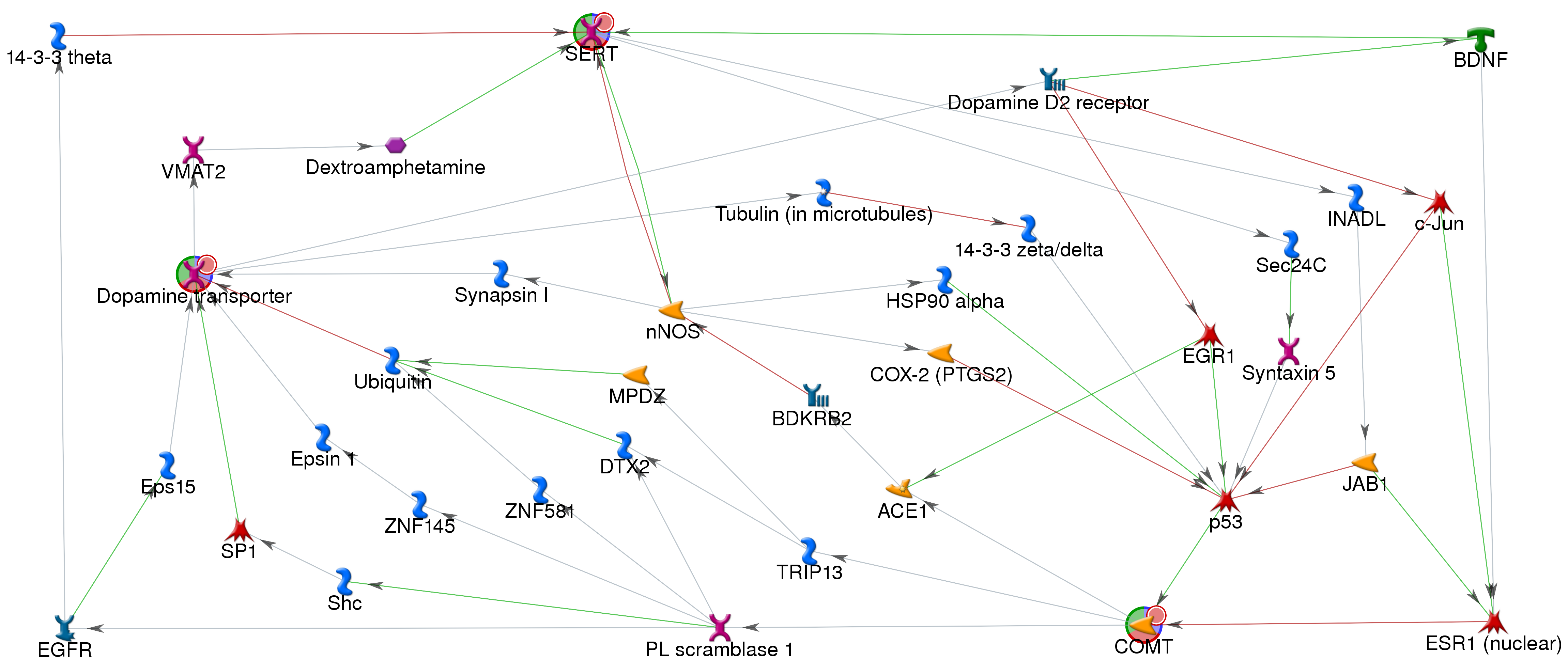

Supplement: Additional file 4 — Figure S1. A Portable Network Graphics file with the GeneGo network that connects the overlapping candidates using the smallest number of nodes at the highest level of confidence for the edges. Overlapping candidate genes (COMT, Dopamine transporter, and SERT) are shown as red, green, and blue circles. Other nodes are coded by the type of protein coded by the gene (e.g. kinases, transporters, etc.). Edges are labeled for direction of effect, where appropriate, and are green for activation or red for repression. [file 1471-2350-11-14-S4.PNG]
